# Examining Nutrition Knowledge, Skills, and Eating Behaviours in People with Severe Mental Illness: A Cross-Sectional Comparison among Psychiatric Inpatients, Outpatients, and Healthy Adults

**DOI:** 10.3390/nu15092136

**Published:** 2023-04-29

**Authors:** Sonja Mötteli, Barbora Provaznikova, Stefan Vetter, Matthias Jäger, Erich Seifritz, Florian Hotzy

**Affiliations:** 1 Department of Psychiatry, Psychotherapy and Psychosomatics, Psychiatric University Hospital Zurich, University of Zurich, 8032 Zürich, Switzerland; 2 University Hospital of Psychiatry and Psychotherapy, University of Bern, 3012 Bern, Switzerland; 3 Psychiatrie Baselland, 4410 Liestal, Switzerland

**Keywords:** severe mental illness, psychiatry, depression, psychosis, health, diet, nutrition knowledge and skills, eating behaviour

## Abstract

Compared to the general population, people with severe mental illness (SMI) have an increased risk of weight gain and metabolic syndrome, but also of malnutrition, in part due to unhealthy lifestyle behaviours. The aim of this cross-sectional study was to identify barriers to healthy eating, including nutrition knowledge and skills in people with SMI. For this purpose, we compared the means of anthropometric data such as body mass index, waist-to-hip ratio, and interview data on nutrition knowledge and skills, health-related variables, eating behaviours, personality, motivation, and attitudes in 65 inpatients and 67 outpatients of the Psychiatric Hospital of the University of Zurich and 64 healthy adults using ANOVA and chi-squared tests. The results showed that patients with SMI had worse nutritional status and lifestyle compared to the healthy controls, including disordered (e.g., night eating) and unhealthy (e.g., high intake of sugary foods) eating habits. However, levels of nutrition knowledge, cooking and food skills, and motivation to eat healthily were not significantly lower in the psychiatric patients than in the healthy adults and were not associated with weight change. Based on our findings, nutritional support for people with SMI is urgently needed and should include not only educational but also behavioural and long-term approaches.

## 1. Introduction

Compared with the general population, people with severe mental illness (SMI), including schizophrenia, major depression, and bipolar disorder [1], often have poorer physical health. They are at higher risk of weight gain and metabolic syndrome, which is associated with an increased risk of diabetes, cancer, and coronary heart disease, as well as a reduced life expectancy [2,3,4,5]. In addition to the side effects of psychiatric medications, such as increased appetite [6,7,8,9], lifestyle factors such as physical inactivity and dietary habits play a crucial role in the pathogenesis of metabolic syndrome in people with SMI [10,11,12]. In general, people with SMI have a poorer diet (higher calorie intake; more processed foods with higher salt and sugar content; less fruit, vegetables, and fibre) compared to the general population [12,13,14,15]. They also have a higher risk of malnutrition with deficiencies in vitamins, minerals, and micronutrients due to inadequate diet [16,17].

People with SMI face several barriers that can lead to unfavourable eating habits and a reduced diet quality [14]. Some of these barriers, such as daily habits, financial constraints, reduced motivation to eat healthily, price, taste, low social support, and the unavailability of healthy foods, have been reported by many people in the general population [18]. Other barriers are more specifically related to psychiatric symptoms and the side effects of psychotropic medication, such as impaired cognition (e.g., problems with planning, memory, and performing nutrition-related tasks), increased appetite and cravings for sweet and fatty foods, lack of daily structure (e.g., unemployment, precarious living conditions), and sedentariness [14,16]. It has also been suggested that people with SMI may have lower levels of nutrition knowledge as well as cooking and food skills. However, research on this topic is limited [14,19,20,21,22]. In people with SMI, such barriers can lead to disordered eating habits, such as eating only one meal per day, night eating, binge eating, fast eating, emotional eating, and continuous snacking, and reduced activities related to planning, shopping, cooking, and eating healthy foods [14,23,24]. In addition, people with SMI often feel bad about their eating behaviours and body weight [25], which may hinder recovery from their mental illness.

The various nutritional challenges and eating problems faced by people with SMI, and the fact that the prevalence of overweight and obesity is two-to-three times higher in this group than in the general population [26], indicate the importance of nutritional support for people with SMI to improve their physical health by reducing the risk of metabolic complications. Indeed, international guidelines strongly recommend the provision of lifestyle interventions in mental health care [27]. However, according to a recent meta-analysis, the effects of dietary interventions on body weight in people with SMI are minimal to moderate [23]. In practice, nutritional support for psychiatric patients is not yet widely available in German-speaking countries [25], although most psychiatric patients believe that nutritional support should be included in standard psychiatric treatment [17]. One reason for this mismatch may be that little is known regarding the important “ingredients” that make a nutritional intervention effective and successful [28].

The present study is a part of a Swiss project to identify and investigate the nutritional needs and challenges of psychiatric inpatients and outpatients with SMI [29]. In a recent publication, the authors showed that most psychiatric patients had experienced weight changes prior to admission (weight gain or loss), and a significant proportion of them were overweight and/or at risk of malnutrition. In addition, one-third of the patients were affected by nutrition-related diseases (e.g., diabetes) or food intolerances, indicating a diversity of nutritional problems in people with SMI [17]. The primary aim of the present study was to examine levels of nutrition knowledge and skills as potential barriers to healthy eating in people with SMI which are often addressed in nutrition counselling and dietary interventions. For this purpose, levels of nutrition knowledge, cooking skills, and food skills, measured by reliable and valid scales, were compared among a group of inpatients, a group of outpatients, and a group of healthy controls. As a secondary objective, several variables related to eating behaviours, such as anthropometric measures, health behaviours, personality, dietary habits, and feelings towards and motivation for healthy eating, were also compared among the groups.

## 2. Materials and Methods

This prospective, cross-sectional study was based on semi-structured interview data and anthropometric measurements from people with SMI treated at the Psychiatric Hospital of the University of Zurich (Psychiatrische Universitaetsklinik Zuerich [PUK]) in Switzerland, including both inpatient (acute wards) and outpatient (day clinic) settings. A control group of healthy adults from the general population of Zurich was also surveyed. The full study is described in a previously published study protocol [29]. Sample 1 included 65 inpatients, Sample 2 included 67 outpatients, and Sample 3 included 64 healthy controls.

### 2.1. Participants and Procedure

Participants were enrolled between September 2021 and August 2022 if they were between 18 and 65 years old, lived in or around the city of Zurich, had sufficient German language skills, were willing to participate in the study, and did not have a diagnosed eating disorder. In addition, the healthy controls were required to have no mental disorders. For the psychiatric samples, clinical staff screened all eligible individuals with psychotic or depressive disorders (classified as F2 or F3 according to the ICD-10 diagnostic tool) upon admission to one of the acute wards of the PUK. At the beginning of their treatment, *n* = 161 inpatients (response rate = 40.4%) and *n* = 94 outpatients (response rate = 71.3%) were consecutively asked to participate in the study. In the case of agreement and the provision of written informed consent, the participant’s contact details were given to the research team. The recruitment process of the psychiatric patients was based on stratified sampling to minimise selection bias and has been fully described and illustrated in a recent publication [17]. For the group of healthy controls, a convenient sample was recruited through various information channels in Zurich, such as flyers in family practice centres, shops, and city swimming pools; university mailing lists; and word-of-mouth advertising among mental health professionals and acquaintances. We used stratified sampling for age and gender for all groups [29,30]. Each interview lasted an average of one hour and was conducted by a specially trained research assistant with background in psychology or medicine. The psychiatric patients were interviewed an average of 14 days after admission. Participants were offered a drink and they were allowed to take breaks according to their needs, but they received no financial compensation. All data were entered into the LimeSurvey tool [31] using case identification codes.

### 2.2. Measures

In addition to nutrition knowledge and skills, a selection of socio-demographic variables, health-related variables, and variables related to the participants’ eating behaviours were included in this study. An overview of all study variables can be found in reference [29].

#### 2.2.1. Personal Variables

The participants’ gender, age, education, nationality, living situation, source of income, and financial situation for food were assessed (see Table 1). Education (high school diploma and above = 1, other = 0) and financial situation ([much] too little = 0, rest = 1) were dichotomised as control variables.

#### 2.2.2. Health Status and Behaviours

Mental health was assessed using the nine-item Symptom Checklist (SCL-K-9) and the nine-item Patient Health Questionnaire (PHQ-9).

The SCL-K-9 is a reliable, efficient, and validated scale for assessing psychopathological symptomatology which asks participants to rate their mental and physical health problems of the past seven days on a five-point Likert-type scale [32]. Higher scores indicate more severe symptoms. The Cronbach’s alpha in the present study was 0.88 (*n* = 196).

The PHQ-9 is a validated and reliable screening tool for assessing the severity of depression in the preceding two weeks [33,34]. Higher scores indicate more depressive symptoms (10–14 = mild depression, 15–19 = moderate depression, 20–27 = severe depression). The Cronbach’s alpha in the present study was 0.89 (*n* = 196).

To quantify the relevance of psychiatric medication on potential weight gain, a sum score of the prescribed psychiatric medications was calculated, with each individual score derived from the clinical information system (higher scores = negative effect on weight gain). Details of the score calculation procedure have been described elsewhere [17].

Participants were asked whether they had received any inpatient or outpatient treatment because of poor physical or mental health in the previous three to six months. Participants were also asked about their smoking status and to report the number of physically active days in a typical week (at least 30 min of moderate-to-vigorous physical activity).

To assess body mass index (BMI; kg/m^2^) and waist-to-hip ratio (WHR; abdominal girth/hip girth), weight (kg), height (m), abdominal girth (cm), and hip girth (cm) were measured using a digital scale (Soehnle 63850 PWD Style Sense Compact 100) and a SECA measuring tape. Each measurement was repeated twice, and the mean was calculated. Participants wore light clothes and no shoes. In addition, participants were asked whether they had lost or gained weight (kg) in the last three to six months and to subjectively rate their nutritional status (supply of energy and nutrients) on a scale ranging from 1 = very poor to 10 = very good. Furthermore, participants were also asked whether they suffered from nutrition-related diseases such as obesity, diabetes, coeliac disease, food allergies, and (subjective) food intolerances, as well as whether they had ever received nutrition counselling.

The validated short form of the Big Five Inventory (BFI-K) was used to assess five personality factors of the participants by rating 21 statements on a Likert-type scale from 1 = strongly disagree to 5 = strongly agree [35]. The Cronbach’s alphas (*n* = 196) were as follows: 0.82 for Extraversion, 0.56 for Agreeableness, 0.70 for Conscientiousness, 0.81 for Neuroticism, and 0.74 for Openness to experience.

#### 2.2.3. Dietary Behaviours and Attitudes

As there are currently no specific dietary assessment tools available for mental health settings [15], we used a selection of simple food frequency questions that have been validated for the general Swiss population and have been shown to be indicators of healthy or unhealthy eating behaviours [36,37]. Participants were asked how often they eat/drink vegetables and salad, fruit, juice, sweets, and sugary drinks (daily, 4–6 times/week, 1–3 times/week, 1–3 times/month, or rarely) and how many portions they usually eat (1–6 handfuls). To compare the intake of sugary foods among the groups, we added up the portions of juice, sweets, and sugary drinks consumed per week.

Participants were also asked to report their usual types of daily meals (e.g., breakfast, morning snack, lunch, afternoon snack, dinner, other snacks between meals), whether they followed a special diet (e.g., gluten-free, lactose-free, low in sugar, high in protein), and whether they intentionally restricted their food intake to regulate weight or health.

Selected items from the Social Functioning Scale [38], specifically developed and validated for use in people with SMI, were used to assess participants’ eating habits. Thereby, participants were asked to indicate, on a scale from 1 = never to 4 = often, how often they performed a particular activity, such as planning or buying food, storing food, preparing meals (food-related activities), eating a healthy and balanced diet, eating prepared meals (healthy eating), eating too little, eating too much (overeating), cooking together with friends, and eating together with friends (social eating).

Participants’ feelings about nutrition were assessed using the technique of spontaneous associations with the words “nutrition” and “diet” [39,40]. In the first step, the participants were asked to name the first words or images that came to mind when they thought of the word “nutrition” or “diet”. In the second step, the participants rated their associations on a scale ranging from 1 (very negative) to 7 (very positive). We then calculated the mean of the ratings. In addition, the participants rated the importance of a healthy and balanced diet to them on a scale from 1 to 10, with higher scores indicating greater importance.

#### 2.2.4. Nutrition Knowledge and Skills

Levels of nutrition knowledge (objective measure) as well as cooking and food skills (subjective measures) were assessed using the sum scores of two different questionnaires.

Nutrition knowledge was assessed using the “practical nutrition knowledge about balanced meals scale” (PKB-7 scale) which has been validated for the Swiss population and used in several countries [37,41,42]. The scale consists of seven multiple-choice items (with only one correct answer) of different levels of difficulty. It aims to measure knowledge relevant to making healthy food choices.

Cooking and food skills were assessed using the Cooking and Food Skills questionnaire [43], previously validated for the Swiss population [44]. The questionnaire includes ratings of confidence in 14 cooking tasks, such as “preparing and cooking vegetables”, and confidence in 19 food tasks, such as “planning how much food to buy”. Confidence ratings were made on a scale from 1 = very poor to 7 = very good and 0 = never/rarely done. The Cronbach’s alpha was 0.89 for the cooking scale and 0.86 for the food scale (*n* = 196).

### 2.3. Statistical Analyses

A sample size calculation suggested the inclusion of at least 192 participants (*n* = 64 per group); for details, see the previously published study protocol [29]. Sociodemographic variables are presented as frequencies by group in Table 1. Chi-squared tests or ANOVA were performed for the variables of participants’ health status and behaviours, dietary behaviours, attitudes, and nutrition knowledge and skills among the inpatients, outpatients, and healthy adults. Where appropriate, post-hoc tests were performed using the Bonferroni correction. In addition, differences in participants’ dietary behaviours, attitudes, and nutrition knowledge and skills were controlled for using ANCOVA with education and financial situation as control variables. Relationships between variables were assessed using Spearman correlation coefficients. In addition, an exploratory extreme-group analysis was performed between psychiatric patients who had experienced weight gain and those who had experienced weight loss using independent samples *t*-tests. The significance level was set at 0.05 for all analyses.

## 3. Results

### 3.1. Characteristics of Participants

A total of 196 individuals took part in the interviews. Table 1 shows the sociodemographic characteristics of the participants, differentiated according to the study groups. The mean age of the study participants was 38.7 (SD = 11.9) years. The group of healthy adults were more highly educated and had better financial situations than the groups of psychiatric patients.

### 3.2. Health Status and Behaviours

The participants’ health status and behaviours differed significantly between psychiatric patients and healthy controls (see Table 2 and Table 3). By definition, the patients had more psychiatric symptoms, had used more medications, and had used medical treatments more frequently in the past few months. There were also significant differences in four of the five personality factors. The psychiatric patients had a higher BMI and WHR and suffered more from nutrition-related diseases and food intolerances compared to the healthy controls. In addition, most patients with SMI had experienced weight changes in the previous three to six months, whereas the healthy controls had more stable weights. In addition to poorer nutritional status, psychiatric patients were less likely to engage in healthy behaviours such as physical activity and not smoking.

### 3.3. Dietary Behaviours, Attitudes, and Nutrition Knowledge and Skills

Table 4 shows the differences in dietary behaviours, attitudes, and nutrition knowledge and skills among the three groups. The psychiatric patients ate more sugary foods and tended to skip main meals when eating late snacks or at night. Higher intake of sugary foods was positively associated with unhealthy behaviours such as late snacking (rs = 0.24, *p* = 0.006, *n* = 132), night eating (rs = 0.17, *p* = 0.046, *n* = 132), and overeating (rs = 0.19, *p* = 0.033, *n* = 132); moreover, this behaviour was negatively associated with healthy behaviours such as eating an individualised diet (rs = −0.23, *p* = 0.008, *n* = 132) and a healthy diet (rs = −0.18, *p* = 0.038, *n* = 132).

The psychiatric patients also performed fewer nutrition-related activities and ate with others less often than the healthy controls. They also had more negative feelings about nutrition, which correlated with the SCL-K-9 scores (rs = −0.32, *p* < 0.001, *n* = 132), the PHQ-9 scores (rs = −0.34, *p* < 0.001, *n* = 132), and social eating (rs = 0.20, *p* = 0.020, *n* = 132).

Although the psychiatric patients had a higher BMI and more nutrition-related diseases and food intolerances (especially outpatients), the proportion of people who ate an individualised diet based on their needs or restricted calories did not differ among the three groups. In contrast to the unhealthier eating behaviours, motivation for a healthy and balanced diet as well as nutrition knowledge and skills were not significantly lower in the psychiatric patients compared to the healthy controls. More precisely, levels of nutrition knowledge and skills were highest in the outpatient group. In addition, participants living in institutions or who were homeless (*n* = 7) reported lower levels of cooking skills (Md = 29.0) and food skills (Md = 54) than those living independently; however, they had similar levels of nutrition knowledge (Md = 5.0).

### 3.4. Differences between Psychiatric Patients with Weight Gain and Loss

**Figure 1:**
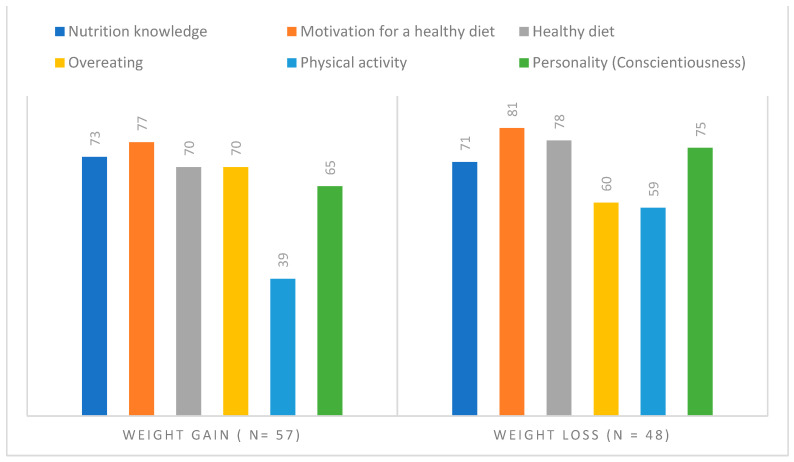
Differences (% of scale scores) in psychiatric patients with weight gain or weight loss.

In an exploratory extreme-group analysis, we compared study variables between psychiatric patients who had experienced weight gain (*n* = 57) in the previous three to six months and those who had experienced weight loss (*n* = 48) during this time to explore differences in health behaviours and attitudes. Sociodemographic variables (e.g., gender, age, education), illness-related variables (e.g., diagnosis, mental state, medication), motivation for healthy eating, and nutrition knowledge and skills were not associated with weight gain or loss. Participants with weight gain had a BMI = 28.7, and those with weight loss had a BMI = 24.7 (t [103] = 4.18, *p* < 0.001), while there was no significant difference in WHR. Weight gain was associated with less physical activity (2.7 vs. 4.1 physically active days per week, t [103] = 3.06, *p* = 0.003), less healthy eating (2.4 vs. 2.8 scale scores, t [103] = 2.40, *p* = 0.018), more overeating (2.8 vs. 2.4, t [103] = 2.48, *p* = 0.015), higher consumption of sweets (11.6 vs. 7.1 servings per week t [103] = 2.14, *p* = 0.035), and lower consumption of vegetables and fruits (25.3 vs. 31.1 servings per week, t [103] = 1.86, *p* = 0.066). In addition, 60% of the participants with weight loss reported eating an individualised diet according to their needs, compared to 33% of those with weight gain (X [1] = 7.70, *p* = 0.006). Weight loss was also associated with higher levels of the following items: Extraversion (2.9 vs. 3.3, t [103] = 1.88, *p* = 0.063), Conscientiousness (3.2. vs. 3.8, t [103] = 3.18, *p* = 0.002), and Openness (3.8 vs. 4.2, t [97.5] = 2.79, *p* = 0.006). Differences in selected variables are presented in Figure 1 as percentages of the scale scores (100% = highest possible scale score).

## 4. Discussion

Patients with SMI are known to be at risk for nutritional problems, mostly associated with increased weight but also malnutrition, leading to reduced life expectancy and quality of life [2,3,4,5,14,16,17]. In the present study, the levels of nutrition knowledge and skills for healthy eating were investigated in individuals with SMI compared to healthy controls, along with the participants’ physical and mental health status and eating behaviours. The results showed that patients with SMI had poorer nutritional status and lifestyle compared to healthy adults. In particular, a substantial proportion of the patients reported disordered and unhealthy eating habits, reduced activities related to food preparation and eating, and negative feelings about nutrition. Despite various nutritional problems, less than half of the participants with SMI followed an individualised diet. However, levels of nutrition knowledge and skills and motivation to eat healthily were not lower in the psychiatric patients than in the healthy controls.

Some previous studies have discussed a lack of nutrition knowledge, cooking and food skills, and motivation to follow a healthy diet as possible causes of unhealthy eating behaviours in people with SMI [14,19,20,21,22]. However, to the best of our knowledge, our study is the first to systematically investigate levels of nutrition knowledge and skills in people with SMI. Contrary to previous assumptions, and despite the higher levels of education of the healthy controls (and likely greater interest in nutrition due to convenience sampling), nutrition knowledge was not higher in the healthy controls. In addition to objectively assessed practical nutrition knowledge, subjective levels of cooking and food skills were not lower in the psychiatric patients than in the healthy controls. More specifically, the inpatients had similar scores to the healthy controls, while the outpatients had the highest scores. One reason for this may be that half of the psychiatric patients (49%) had already received dietary advice. There may be two reasons for the differences between the inpatients and outpatients. First, because of their higher BMI and poorer nutritional status, the outpatients may have been more interested in gaining knowledge about a healthy diet. Second, some of the inpatients who lived in residential care homes (including one homeless person) were unable to prepare food by themselves and therefore had very low cooking and food skills. In addition to nutrition knowledge and skills, motivation to eat healthily was not significantly lower in the psychiatric patients than in the healthy controls. Moreover, levels of knowledge, skills, and motivation were not related to weight gain or weight loss in the psychiatric patients.

Nevertheless, the psychiatric patients seemed to have difficulty translating their knowledge into action. In line with previous findings [10,11,12,24], they had overall unhealthier lifestyles compared to the healthy controls, such as higher smoking rates, lower physical activity levels, and unhealthy and disordered eating habits, such as higher consumption of sugary foods, skipping main meals, night eating, less social eating, and generally less nutrition-related activities such as planning meals or shopping for food. Patients with SMI often have disturbed circadian rhythms and a lack of daily structure (e.g., unemployment). Both diagnoses included in the study (psychosis and depression) can be associated with disordered sleep patterns [45]. The combination of SMI, sleeplessness, and social deprivation may lead to night eating and reduced social eating and activities during the day [46]. This is associated with higher calorie intake, problematic aspects of body metabolism, and a higher risk of metabolic syndrome [47]. Furthermore, social aspects of eating are associated with feelings of inclusion and psychological well-being and may lead to preferable food choices and healthier eating patterns [48,49]. Indeed, in an implicit association task, the patients expressed more negative feelings towards nutrition than the healthy controls.

Aside from lifestyle factors, increased appetite and weight gain in patients with SMI are often discussed as being caused by side effects of psychiatric medication [23]. For instance, second-generation antipsychotics and some antidepressants are known to lead to increased appetite. In our study, most patients had medications with a known risk of metabolic side effects. The inpatients with 62% affective diagnoses (F3) had a higher sum score, which entails a higher risk for weight gain than outpatients with 87% affective diagnoses [17]. However, despite a higher medication-induced risk for weight gain in the inpatients and the need to prevent metabolic side effects of psychiatric medication [6,7,9], BMI was significantly higher in the outpatients, which indicates the importance of lifestyle factors. Similarly, the results of the exploratory extreme-group analysis showed positive associations between an unhealthy lifestyle including low physical activity, unhealthy eating patterns (high sugar as well as low vegetable and fruit consumption), and weight gain. In contrast, patients who reported weight loss during the past three to six months were more physically active, followed a healthy and individualised diet, and were characterised by more conscientiousness, extraversion, and openness. Some personality traits seem to facilitate or hinder the adoption of a healthy lifestyle in patients with SMI, and strategies to increase levels of conscientiousness have been discussed to achieve desirable behaviours and outcomes in psychotherapy [50]. However, their specific role in eating behaviours must be explored in future studies.

A strength of this study is the use of a broad range of different objective (e.g., BMI, nutrition knowledge) and subjective measures (e.g., weight change, cooking and food skills) related to nutrition being applied in patients with SMI. Thus, we can assume that biases based on subjective assessments and social desirability were not substantial. As well, we used a complete stratified sampling frame for the recruitment of the patients, meaning that each patient meeting the inclusion criteria had the chance to participate during the recruitment period, thus minimising selection biases. However, this was not the case for the convenience sample of the healthy controls. The patient groups and healthy controls differed in terms of their education and socioeconomic status. Nevertheless, these gaps have been frequently described [51] and might also represent the naturalistic setting. Another limitation of this study is the use of a cross-sectional design, which was appropriate for our research aims but did not allow us to examine changes and causal relationships.

## 5. Conclusions

In summary, the results of this study showed that the participants with SMI had worse mental and physical health conditions compared to the healthy adults. The patients with SMI were frequently affected by nutrition-related diseases such as overweight and diabetes and more often had disordered eating habits (e.g., night eating) and unhealthy lifestyle and eating patterns (e.g., low physical activity, smoking, high intake of sugary foods). Thereby, nutrition knowledge, cooking and food skills, as well as motivation for a healthy diet did not seem to be relevant barriers for these behaviours, although they might be important prerequisites for healthy eating. Rather, the results indicated that people with SMI would benefit from nutritional support that aims at improving their daily structure and social inclusion, for instance via behavioural approaches related to meal planning and social eating. Therefore, the development of low-threshold, long-term support services are needed in addition to individual nutrition counselling. Based on the study’s results, there might also be different needs for support services in inpatient and outpatient settings as well as in terms of living situation (e.g., living alone or in a residential care home). In addition, the results indicate a need for more knowledge about how food intolerances and personality factors affect the eating behaviours of people with SMI.

## Figures and Tables

**Table 1:** Characteristics of participants.

Sociodemographic Variables	Psychiatric Inpatients (*n* = 65)		Psychiatric Outpatients (*n* = 67)		Healthy Adults (*n* = 64)	
	*n*	%	*n*	%	*n*	%
Gender, female	32	49	34	51	32	50
Age > 40 years	32	49	34	51	32	50
Swiss nationality	46	79	45	79	46	79
Education						
Compulsory schooling	14	22	9	13	2	3
Vocational education	22	34	30	45	15	23
Matura (high school exit exam)	7	11	6	9	5	8
Higher vocational education	8	12	8	12	21	33
University	14	21	14	21	21	33
Source of income						
Salary	21	32	32	47	52	81
Disability pension	18	28	5	8	0	0
Social-welfare benefits	12	18	15	22	2	3
Support by family	9	14	10	15	9	14
Savings	3	5	5	8	0	0
Unknown	2	3	0	0	1	2
Financial possibilities for nutrition						
Far too little	5	8	10	15	0	0
Too little	4	6	10	15	2	3
Just sufficient	15	23	15	22	6	9
Adequate	41	63	32	48	56	88
Housing situation						
Alone	21	32	29	43	13	20
Together with others	37	57	38	57	51	80
Residential care home	6	9	0	0	0	0
Homeless	1	2	0	0	0	0

Note. N total = 196, *n* = 23 missing values in the variable Swiss nationality.

**Table 2:** Participants’ health status and behaviours (continuous variables).

Variables	Inpatients (*n* = 65)		Outpatients (*n* = 67)		Healthy Adults (*n* = 64)			
	M	SD	M	SD	M	SD	F	*p*
SCL-K-9	1.8 a	0.8	2.0 a	0.9	0.7 b	0.5	54.41	<0.001
PHQ-D	13.5 a	6.3	16.3 b	5.1	4.5 c	3.3	96.47	<0.001
Sum score of prescribed psychiatric medication	2.9 a	2.7	1.6 b	2.0	0.0 c	0.0	37.67	<0.001
Personality (Big 5 factors)								
Extraversion	3.4	1.1	2.9	0.9	3.8	0.8	15.21	<0.001
Compatibility	3.3	0.8	3.1	0.8	3.5	0.7	4.64	0.011
Conscientiousness	3.7	0.7	3.2	0.9	3.8	0.6	9.52	<0.001
Neuroticism	3.4	0.9	3.9	0.8	2.4	0.8	51.73	<0.001
Openness	4.0	0.7	3.9	1.0	3.8	0.7	1.17	0.313
Physical activity (number of days per week)	3.4 ab	2.4	3.0 a	2.6	4.3 b	1.9	5.27	0.006
Subjective evaluation of nutritional status	6.6 a	2.3	5.5 b	2.2	8.2 c	1.5	29.18	<0.001
BMI (kg/m^2^)	25.3 a	5.0	27.9 b	5.3	24.0 a	3.7	11.50	<0.001
WHR (cm)	91.27 a	8.0	89.62 ab	10.6	85.78 b	11.0	5.20	0.006

Note. M = mean, SD = standard deviation. For continuous data, ANOVA was calculated; difference in letters (a,b,c) indicate significant Bonferroni-adjusted post-hoc tests, *p* = 0.05. SCL-K-9: higher scores = more psychiatric symptoms; PHQ-D: higher scores = more depressive symptoms; sum score of prescribed psychiatric medication: higher scores = negative effect on weight gain; personality factors: higher scores = higher expression; subjective evaluation of nutritional status: 1 = very bad, 10 = very good.

**Table 3:** Participants’ health status and behaviours (binary variables).

Variables	Inpatients (*n* = 65)		Outpatients (*n* = 67)		Healthy Adults (*n* = 64)			
	*n*	%	*n*	%	*n*	%	Pearson Chi-Square	*p*
Experienced weight change in last 3–6 months							38.72 (4)	<0.001
Weight gain	18 a	28	39 b	58	17 a	27		
No change	19 a	29	8 b	12	36 c	56		
Weight loss	28 a	43	20 ab	30	11 b	17		
Nutrition-related diseases (e.g., diabetes, celiac disease, food allergies)	12 ab	19	24 a	36	11 b	17	7.86 (2)	0.020
Food intolerances	21 ab	32	34 a	51	15 b	23	11.12 (2)	0.004
Smokers	21 a	60	35 a	52	13 b	20	23.12 (2)	<0.001
Medical treatment due to mental problems in last 3–6 months	21 a	97	67 a	100	3 b	5	196.11	<0.001
Medical treatment due to physical problems in last 3–6 months	21 a	62	40 a	60	16 b	25	21.91 (2)	<0.001
Previous experience with nutrition counselling	21 a	46	34 a	51	11 b	17	18.17 (2)	<0.001

Note. For nominal data, a chi-squared test was calculated; and if *n* < 5 the Fisher–Freeman–Halton Exact Test was used. Difference in letters (a,b,c) indicate significant Bonferroni-adjusted post-hoc tests, *p* = 0.05.

**Table 4:** Participants’ dietary behaviours, attitudes, and nutrition knowledge and skills.

Variables (Possible Scale Range)	Inpatients (*n* = 65)		Outpatients (*n* = 67)		Healthy Adults (*n* = 64)			
	*n* or M	% or SD	*n* or M	% or SD	*n* or M	% or SD	F or Pearson Chi-Square	*p*
Food intake								
Portions of vegetables and salad per week	17.0	11.5	14.1	11.0	20.6	12.7		
Portions of fruits per week	8.9	7.5	7.3	7.4	7.7	7.3		
Portions of juice per week	4.6	6.1	2.6	4.8	2.3	3.6		
Portions of sweets per week	7.1	7.8	10.6	12.0	8.8	8.6		
Portions of sugary drinks per week	6.1	9.3	9.5	13.9	2.5	5.1		
Portions of sugary food (juice, sweets, drinks) per week	17.8	16.6	22.7	18.8	13.6	12.3	5.22	0.006
Meal structure								
Breakfast	46	71	35	52	43	67		
Mid-morning snack	20	31	11	16	15	23		
Lunch	56	86	46	69	61	95		
Afternoon snack	18	28	24	36	22	34		
Dinner	63	97	58	87	61	95		
Late snack	22	34	29	43	17	27		
Night eating	16 a	25	17 a	25	5 b	8		
Sum of main meals (0–3)	2.5 a	0.7	2.1 b	0.7	2.6 a	0.6	11.57	<0.001
Diet								
Individualised diet (e.g., gluten-free, lactose-free, low in sugar, rich in protein, etc.)	28	43	29	43	36	56	2.95	0.228
Dieting (caloric restriction due to weight or health)	26	40	36	54	35	55	3.52	0.172
Eating habits (1–4)								
Nutrition-related activities	3.1 a	0.7	3.1 a	0.7	3.5 b	0.6	7.27	<0.001
Healthy eating	3.0 ab	0.8	2.8 a	0.7	3.1 b	0.6	3.76	0.025 *
Overeating	2.6	0.8	2.6	0.8	2.9	0.6	2.86	0.060
Social eating	2.6 a	0.8	2.2 b	0.9	3.1 c	0.8	20.38	<0.001
Attitudes								
Implicit feelings towards nutrition (1–7)	5.4 ab	1.4	5.0 b	1.5	5.9 a	1.2	6.46	0.002
Importance of eating a healthy and balanced diet (1–10)	8.2	1.8	8.0	2.0	8.6	1.4	2.49	0.086
Knowledge and skills								
Practical nutrition knowledge (0–7)	4.5 a	1.6	5.3 b	1.5	5.0 ab	1.5	4.53	0.012
Confidence in cooking skills (0–98)	50.6 a	25.0	65.7 b	23.0	60.9 b	21.1	7.27	<0.001
Confidence in food skills (0–133)	64.6 a	27.7	76.4 b	24.8	74.6 ab	25.1	3.95	0.021

Note. M = mean, SD = standard deviation. For continuous data, ANOVA was calculated; for nominal data, a chi-squared test was calculated; and if *n* < 5, the Fisher–Freeman–Halton Exact Test was used. Difference in letters (a,b,c) indicate significant Bonferroni-adjusted post-hoc tests, *p* = 0.05; higher sum scores mean higher frequency of habits, more positive feelings, higher importance, more knowledge and skills; * adjusted for education and financial situation; *p* = 0.519 (all other results remained stable).

## Data Availability

The dataset generated for the current study is available from the first author (S.M.) upon written request.
